# The Prevention and Cure of Tuberculosis

**Published:** 1896-10-24

**Authors:** 


### The Hospital

A Journal of

The Medical Sciences and Hospital Administration, with

AN EXTRA NURSING SUPPLEMENT.

Vol. XXI.—No. 526.] [Oct. 24, 1896.

### The Prevention and Cure of Tuberculosis.

A discussion by some of the most representative members of the medical profession in Great Britain on the important subject of tuberculosis, which was opened by Dr. John William Moore in an excellent paper read before the last annual meeting of the British Medical Association, enables us to focus knowledge on this particular question with great precision, and to make practical deductions of an exceedingly important character. To begin with, it is generally held that consumption in our times claims fewer victims than it formerly did; and this, at any rate so far as England, Scotland, and Wales are concerned, is quite true. Ireland, on the other hand, for what reason does not clearly appear, lags decidedly behind in this scientific "forward movement." But though it is undoubtedly the case that the statistics of tuberculosis, both as to prevention and as to cure, show a decided improvement in England, Scotland, and Wales, we are, nevertheless, still compelled to admit the appalling facts that of all the deaths registered in England and Wales during the fourteen years 1881-1894, no fewer than 12·3 per cent. were due to this disease alone, whilst in Scotland a still larger proportion, 13·8 per cent., were caused by it. In Ireland, as we have already stated, there is little or no improvement to record; and during the fourteen years under consideration 14·9 per cent., or about one in every seven of all registered deaths, were due to tuberculosis. One more fact, for purposes of comparison, may be stated, namely, that in the City of Brussels, one of the most healthily situated of all European capitals, nearly one quarter, or 23·1 per cent., of all the deaths registered in the twenty years immediately preceding 1890 were owing to this cause alone. There is thus, notwithstanding a decided improvement in our own country, a state of things still existing with regard to tuberculosis which can hardly be admitted to be anything less than a scandal to European civilisation.

Now, ill-informed persons are apt to lay all facts of this kind at the door of the medical profession. But the scandal, which is of a truly monstrous order, cannot be charged upon medical science; it is civilisation, State authority, general opinion, which are at fault. What medicine can do that she has done. She has investigated the disease and its ætiology so thoroughly that now she is able to say, with absolute certainty, exactly what is its cause, exactly how it spreads among and kills its victims, exactly how it may be prevented, and, in many cases, exactly how it can be cured. And if all this knowledge is not utilised, it is not because it has not been amassed, nor is it because medicine has kept it to herself. She has published it again and again, as from the housetops.

We may be challenged on the question of prevention. But a clear understanding of the source and origin of tuberculosis makes clear at once the scientific possibility of its prevention. Tuberculosis is a bacillary disease. The bacillus, whose entrance and multiplication within the body are of such deadly import, can be destroyed with comparative ease without the body. The complete isolation of all consumptive persons, with the destruction of their expectorations, and the non-use of tuberculous milk and meat, ought to result in a large diminution of the disease in any given country. In order to its complete extirpation, there must, of course, be wider and more detailed sanitary measures employed than these. We quite see the difficulties in the way of such widespread measures, which really mean a raising of the whole standard of comfort accepted by the people; but when it is considered how important a step the mere destruction of tubercle bacilli is in the prevention of tuberculosis, and that in many cases such destruction is well within the means of a really practical science, it does make the scientific writer feel a little ashamed of his country when he sees so little effort made to prevent consumption from carrying off its victims.

In discussing the treatment of tuberculosis, as at present practised by the most responsible members of the profession, we can by no means dogmatise so confidently. Nevertheless, even in treatment, not only have great advances been made, but, provided persons predisposed, and persons already infected, be seen and treated in the very early stages of impaired health, it is wonderful how many and what permanent cures can be effected. The predisposed, Dr. Moore tells us, should recognise their predisposition, and so should their doctors,

In this every physician who joined in the discussion entirely agreed with Dr. Moore. Briefly summarising the treatment of the predisposed, it consists largely in intelligent attention to the "diet and the dwelling-house." A generous, easily assimilated diet, with strict attention to regularity in meals, is indispensable, whilst the dwelling-house should be "well built, well sewered, well ventilated, and standing on high and dry ground in the open country, on mountain slopes, or near, but not too near, the sea shore." If it be objected that all this is possible only for the few, the reply is that the few who are able may provide the conditions for themselves, whilst for the many who are unable some effort should be made to build special hospitals on specially selected sites. If a special hospital be ever justified it is justified on almost every conceivable ground in the case of consumption, or those who are threatened with consumption.

On the question of the cure of patients already advanced in phthisis, great caution was observed by all who took part in the discussion. But although no specific for the cure of advanced phthisis has yet been or can be announced, a resolution of great practical importance was agreed to. The resolution decided in favour of bacteriological laboratories, in which diagnosis could be bacteriologically demonstrated, at the public expense; and also that persons of the less wealthy classes suffering from advanced consumption ought to be treated and isolated at the public cost, or by charitable agencies. The first result of this discussion is, that whilst established tuberculosis is very difficult to cure, the disease is comparatively easy of prevention; and the obvious corollary is that, if so deadly an enemy can be destroyed before it does any harm, public duty demands that it shall be so destroyed by all available means.

